# Assessment of field performance and bruchid resistance during seed storage of a genetically modified cowpea expressing the alpha-amylase inhibitor 1 protein from common bean

**DOI:** 10.3389/fpls.2024.1478700

**Published:** 2024-11-22

**Authors:** Jerry A. Nboyine, Muhammad L. Umar, Gloria A. Adazebra, Iliyasu M. Utono, Philip Agrengsore, Frederick J. Awuku, Mohammed F. Ishiyaku, Jose M. Barrero, Thomas J. V. Higgins, Donald J. MacKenzie

**Affiliations:** ^1^ Entomology Section, Council for Scientific and Industrial Research, Savanna Agricultural Research Institute, Tamale, Ghana; ^2^ Intitute for Agricultural Research, Ahmadu Bello University, Zaria, Nigeria; ^3^ Council for Scientific and Industrial Research, Savanna Agricultural Research Institute, Tamale, Ghana; ^4^ Commonwealth Scientific and Industrial Research Organisation, Agriculture and Food, Canberra, ACT, Australia; ^5^ Institute for International Crop Improvement, Donald Danforth Plant Science Center, St. Louis, MO, United States

**Keywords:** alpha-amylase inhibitor, bruchids, cowpea, genetic modification, storage losses, host plant resistance

## Abstract

**Introduction:**

The cowpea weevil, *Callosobruchus maculatus* Fab., is the most economically important storage pest of cowpeas, causing up to 100 percent grain losses within six months of storage. To sustainably resist weevil damage, the cowpea cultivar IT86D-1010 was genetically modified via *Agrobacterium*-mediated transformation to produce event CSI-32, which expresses the kidney bean alpha-amylase inhibitor 1 (αAI-1) protein exclusively in the seed, providing suppression of weevil development.

**Methods:**

Trials were conducted in Ghana and Nigeria during the 2023 growing season to assess the performance in the field and in post-harvest storage of this transgenic event (CSI-32) and of four check lines: the non-transformed parental line (IT86D-1010) and three released varieties (SAMPEA 7, SAMPEA 20-T and IT13K-1070-2). Data collected from the field trials comprised plant growth parameters, pest infestations and damage, and grain yield. Harvested grain from each replicated entry was used in a storage assessment of bruchid resistance following artificial infestation with laboratory-reared cowpea weevils. Data were collected on egg oviposition, adult emergence, and grain damage as well as computation of median development period and Dobie’s susceptibility index for each entry.

**Results and discussion:**

The agronomic performance and phenotypic characteristics of event CSI-32 were very similar to its parental counterpart and the other compared varieties. However, event CSI-32 exhibited complete suppression of weevil emergence and resistance to seed damage over the four-month period of the post-harvest study.

**Conclusions:**

This work represents the first field study of genetically modified cowpea expressing the αAI-1 protein. It demonstrates how a biotechnology solution to mitigate significant economic losses during cowpea storage, offers great potential for cowpea improvement programs.

## 1 Introduction

Cowpea, *Vigna unguiculata* (L.) Walp., is a drought-tolerant and warm weather leguminous crop that is well adapted to the drier regions of the tropics (Singh, 2005). The grains of this legume are widely consumed and serve as an essential source of plant protein to millions of people in sub-Saharan Africa (SSA) and other parts of the world (Bolarinwa et al., 2022; Ige et al., 2011; Nwagboso et al., 2024). The grains are severely damaged during storage by a bruchid, the cowpea weevil, *Callosobruchus maculatus* Fab. (Coleoptera: Chrysomelidae) (Hajam and Kumar, 2022; Devi and Devi, 2014). Infestation starts on the field when females get into damaged or shattered pods and lay their eggs on the seeds. Under favourable environmental conditions in storage, these eggs hatch and the weevil population builds up rapidly leading to both qualitative and quantitative grain losses (Singh et al., 1985; Kpoviessi et al., 2019; Torres et al., 2016). Weevil damage is manifested as reductions in seed weight and germination ability, reduced nutritional value, and adverse effects on the appearance of cowpeas, rendering them unfit for consumption and commercialization (Torres et al., 2016). In general, losses due to cowpea weevil infestation can be as high as 100 percent within six months of storage of untreated cowpea (Mkenda et al., 2014).

To effectively mitigate losses due to this pest, the use of hermetic technology, such as the Purdue Improved Crop Storage (PICS) bags, has proved to be an environmentally-friendly strategy (da Silva et al., 2018). Thermal disinfection has also been reported to be effective at managing eggs, larvae, pupae, and adults of this insect although the different stages require different temperatures and different times of exposure (Loganathan et al., 2011). Other environmentally benign strategies include the use of botanicals (*Eugenia aromatica* (Baill.), *Cymbopogon citratus* (lemon grass), *Citrus sinensis* (orange peel) and *Azadirachta indica* (neem) (Ofuya et al., 2010; Ojebode et al., 2016; Kusuma et al., 2014), Spinosad sprays (Sanon et al., 2010), vegetable oils (Khan, 2011), and microbial pesticide formulations containing *Bacillus thuringiensis* (Malaikozhundan and Vinodhini, 2018). These management approaches, however, are not widely adopted by resource-poor farmers and others in the cowpea value chain because of limited access to these protection agents and the high cost of some of these management options. For botanical extracts, the labour associated with their preparations often limit their adoption in SSA, while the price of those industrially prepared and marketed ones are prohibitive.

Hence. most farmers, grain merchants and seed producers rely on synthetic insecticides, such as carbamates (propoxur), organochlorines (lindane), organophosphates (e.g., acephate, diazinon, dichlorvos, dursban, malathion, pirimiphos-methyl), synthetic pyrethroids (permethrin), phosphine and phostoxin, methyl bromide, and iodofenphos, to mitigate losses due to the cowpea weevil (Ekoja et al., 2021; Hajam and Kumar, 2022). Insecticide misuse to control bruchids can be the main cause of harmful pesticide residues often found in marketed cowpea grains (Olufade et al., 2014; Olutona and Aderemi, 2019). Residues of these insecticides in the grains, and the development of insect resistance to the active ingredients in their formulations are major concerns. There is therefore a need to increase the quality of stored cowpea by exploring more effective, economic, and environmentally friendly cowpea weevil management alternatives. The development of varieties with resistance to infestation and damage by the cowpea weevil is the most viable cost-effective method of overcoming this damaging storage pest and increasing access to the grains of this nutritious legume (Singh et al., 1985; Kpoviessi et al., 2019; Nyarko et al., 2022). However, to date there is no known conventionally bred cowpea variety with an adequate level of protection from infestation and damage by this weevil. Only a few varieties with modest resistance under low insect pressure have been identified, and this is usually influenced by physical seed attributes such as surface area, smoothness, and curvature (Adam and Baidoo, 2008; Ajayi and Lale, 2001; Musa and Adeboye, 2017), as well as chemical traits such as production of trypsin, arcelin, and p-aminophenylalanine (Amusa et al., 2014; Badii et al., 2013; Onigbinde and Oigiangbe, 1996; Tengey et al., 2023).

In the absence of adequate sources of resistance in conventional breeding programs, biotechnological approaches are needed to introduce effective and durable protection from cowpea weevil infestation and damage. Starch digestion involves the breakdown by α-amylase to small linear and branched malto-oligosaccharides, which are in turn hydrolyzed to glucose by α-glucosidases. Several insects, especially bruchids that feed on starchy seeds during larval stages, depend on their α-amylases for survival. The α-amylase inhibitor 1 (αAI-1) protein produced in the common bean (*Phaseolus vulgaris*) binds irreversibly to the enzyme active site and inhibits the activity of porcine, human, and insect α-amylases (Altabella and Chrispeels, 1990) and is thus toxic to bruchids in adzuki beans (Ishimoto et al., 1996), peas (Schroeder et al., 1995; Shade et al., 1994), chickpeas (Sarmah et al., 2004), and cowpeas (Solleti et al., 2008; Lüthi et al., 2013). Cowpea line IT86D-1010 was genetically modified via *Agrobacterium*-mediated transformation to create transgenic event CSI-32, which expresses the kidney bean αAI-1 protein exclusively in the seed as a means of controlling post-harvest damage due to the bruchid weevil.

The current study evaluated the phenotypic characteristics and post-harvest resistance to weevil damage of CSI-32 cowpea in comparison with its non-transformed parental line, IT86D-1010, and three released cowpea cultivars, including SAMPEA 7, SAMPEA 20-T and IT13K-1070-2. The purpose of the field phase was to assess whether there were any adverse effects on agronomic performance or plant phenotype arising from the genetic modification resulting in event CSI-32, while the storage study on harvested grain was intended to evaluate the efficacy of the introduced weevil resistance trait.

## 2 Materials and methods

### 2.1 Plant materials

Event CSI-32 was produced as described by Higgins et al. (2012) at the Commonwealth Scientific and Industrial Research Organisation (CSIRO) in 2006. This was done by transforming cultivar IT86D-1010 with a binary vector carrying the kidney bean *αAI-1* gene under the control of the seed-specific phytohemagglutinin PHA-L (*dlec2*) promoter (Higgins et al., 2012). Homozygous T_10_ seeds were multiplied in Canberra, Australia, in 2023 and sent to Ghana and Nigeria for the field trials. The check lines used in these trials were the non-transformed parental line, IT86D-1010, which is derived as a cross between Tvx4659-03E × IT82E-60. The other lines were SAMPEA 20-T (IT97K-499-35 containing transformation event AAT-709AA-4), IT13K-1070-2 (moderately resistant to bruchids), and SAMPEA 7 (highly susceptible to bruchids). Detailed descriptions of the plant material are presented in **Table 1**. Apart from IT86D-1010, which was obtained from CSIRO, the other lines were sourced from the Institute for Agricultural Research (IAR), Ahmadu Bello University (ABU) Zaria, Nigeria.

**Table 1:** Summary of the chemical properties of soils at the confined field trial sites at Nyankpala, Ghana and Zaria, Nigeria.

Soil parameter	Nyankpala, Ghana^1^	Zaria, Nigeria^2^
pH H2O8 (1:2.5)	4.30	4.48
Organic Carbon (%)	0.43	0.41
Nitrogen (%)	0.039	0.037
Phosphorus (mg/kg)	4.65	3.87
Potassium (mg/ka)	48	42
Calcium (Cmol+/kg)	1.4	1.2
Magnesium (Cmol+/kg)	0.6	0.4

Sources: ^1^Soil Analysis Data Sheet, Soil Chemistry Laboratory, CSIR-Savanna Agricultural Research Institute, Nyankpala, Ghana; ^2^Extract of Soil Analysis Data Sheet, Soil Science Department, Institute for Agricultural Research, Ahmadu Bello University, Zaria, Nigeria.

### 2.2 Field studies

#### 2.2.1 Ecology of the study areas

**Figure 1:**
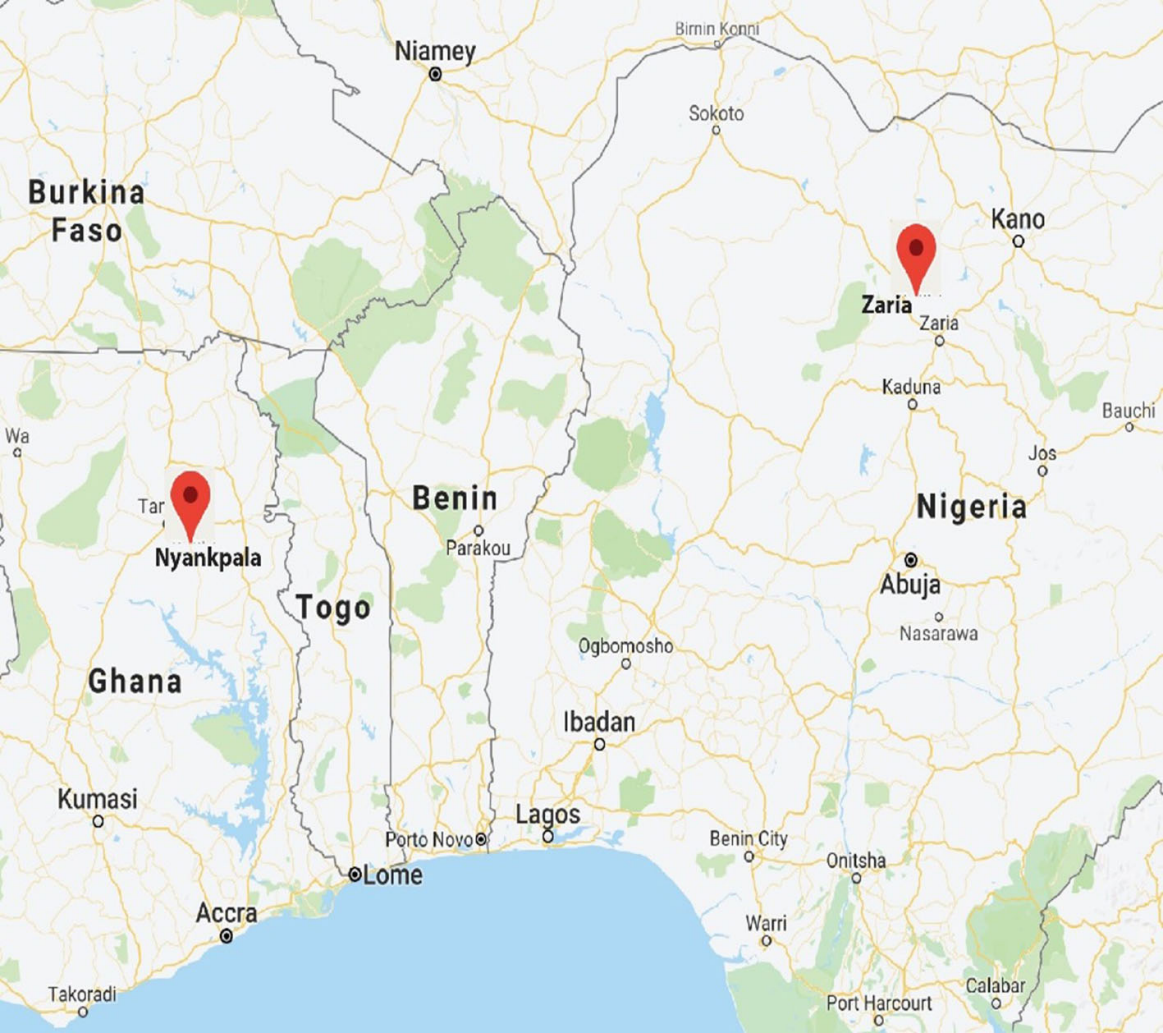
Map showing field trial locations in Ghana and Nigeria during 2023.

Regulated confined field trials (CFTs) were conducted at the authorized CFT sites at CSIR-SARI, Nyankpala, and IAR, Zaria (**Figure 1**). The CFT sites at Nyankpala and Zaria are classified as “Aw” (tropical wet and dry or savanna) climate under the Köppen-Geiger climate classification (Geiger, 1961). Both sites are characterized by unimodal rainfall pattern with the rainfall in Nyankpala commencing in April and ending in October, while that of Zaria starts in June and ends in October. The mean annual rainfall at Nyankpala ranges between 1000 mm and 1200 mm while that of Zaria ranges from 1200 mm to 1600 mm. The mean annual temperature during the growing season ranges from 23°C to 35°C for Nyankpala and 20°C to 36°C for Zaria.

The soil at Nyankpala is sandy loam and it developed from the Voltaian sandstones known as the Nyankpala series (Serno and van de Weg, 1985) (see **Table 1**). In contrast, Zaria soils are leached ferruginous tropical soils with high clay content and overlying drift materials, classified as Typic Haplustalf or Alfisol in the United States Department of Agriculture system (Baillie, 2001; Bekele and Birhan, 2021) (see **Table 1**).

#### 2.2.2 Trial layout and management

At both trial sites, the fields were tractor-ploughed followed by harrowing to obtain a fine soil tilth. The prepared fields were laid out as randomized complete block design (RCBD) after which ridges were manually constructed on each plot at a spacing of 75 cm. The cowpea entries (**Table 2**) were then randomly assigned to the plots in each block. There were six blocks of each entry. At Nyankpala, plots comprised five rows of cowpea planted on ridges that were four meters long, while at Zaria, plots consisted of four rows of cowpea planted on ridges that were five meters long. The intra-row spacing was 20 cm and there were two plants per hill. Plots within a block were separated by 1.5 m wide alleys while blocks were two meters apart.

**Table 2:** Description of entries used for planting.

Entry	Genotype	Pedigree	Description
1	CSI-32	Transformation event derived from IT86D-1010.	Sourced from the Commonwealth Scientific and Industrial Research Organisation (CSIRO), Agriculture & Food, Canberra, Australia. Transgenic line resistant to *C. maculatus* (cowpea weevil). Seed for planting was the T_10_ selfed generation.
2	IT86D-1010	Derived from a cross between Tvx4659-03E × IT82E-60.	An advanced breeding line, medium maturity (71 d), photo-insensitive, with semi-erect growth habit. It has combined resistance to cowpea yellow mosaic, blackeye cowpea mosaic, and many strains of cowpea aphid-borne mosaic, Cercospora, smut, rust, Septoria, scab, Striga, Alectra, aphids, and thrips (Van Boxtel et al., 2000; Lale and Kolo, 1998).
3	SAMPEA 20-T	Derived from SAMPEA 10 (IT97K-499-35) containing transformation event AAT-7Ø9AA-4.	A high yielding, early maturing variety that is also resistant to Striga and Alectra, two notorious parasitic weeds that are a major constraint to cowpea production in most producing areas in Nigeria and other dry savannah regions. This variety expresses the Cry1Ab insecticidal protein and was granted variety registration in Nigeria in December 2019.
4	IT13K-1070-2		A white-seeded variety with black eye, rough coat texture, and medium seed size (14-17 g/100 seed). Tengey et al. (2023} found the variety to be moderately resistant and concluded that it could be used as a source of genes for resistance to bruchid to improve otherwise susceptible genotypes.
5	SAMPEA 7 (IAR 48)		Brown seeded medium maturity variety released in 1986 and registered in Nigeria in 1996. Consistent and stable, high yielding potential and good palatability. The variety is quite susceptible to *C. maculatus* attack (Ojumoola and Adesiyun, 2014).

A starter dose of NPK (15:15:15) fertilizer was applied at one week after planting at a rate of 40 kg/ha (**Table 1**). Weed control was conducted manually at two weeks after planting and at podding. At the early vegetative growth stage, a single round of insecticide [Lambda-Cyhalothrin (15g/l) + Acetamiprid (20g/l)] spray was applied on 6^th^ August to manage aphid infestation in Ghana. In contrast, a systemic and contact fungicide (Carbendazim 12% + Mancozeb 63% W.P.) spray was applied on 15^th^ August to mitigate foliar disease infection at the vegetative growth stage in Nigeria. Afterwards, insecticide sprays were applied weekly from flower bud initiation until pod filling using Lamsate^®^ (Lambda-Cyhalothrin 15g/l + Dimethoate 300 g/l) at a rate of 0.5 l/ha (**Table 3**). These insecticide sprays targeted vegetative stage (*Bemisia tabaci* Genn, *Empoasca*, *Aphis craccivora* Koch), flowering (*Megalurothrips sjostedti* Try., *Maruca vitrata* F.) and podding stage pests such as the pod-sucking bugs complex (*Clavigralla tomentosicollis* Stal, *Anoplocnemis curvipes* (F.), *Riptortus dentipes* (F.), *Nezara viridula* L., *Thyanta custator* (F.). and *Aspavia armigera* L.).

**Table 3:** Dates of application of insecticide with active ingredients comprising Lambda-Cyhalothrin (15g/l) and Dimethoate (300 g/l) at the confined field trial sites at Nyankpala, Ghana and Zaria, Nigeria.

Dates of insecticide applications	
Nyankpala, Ghana	Zaria, Nigeria
6^th^ August, 2023*	15^th^ August, 2023**
30^th^ August, 2023	30^th^ August, 2023
6h September, 2023	6^th^ September, 2023
13^th^ September, 2023	14^th^ September, 2023
20^th^ September, 2023	21^st^ September, 2023
27^th^ September, 2023	28^th^ September, 2023
4^th^ October, 2023	4^th^ October, 2023

*= a single round of spraying using insecticide with Lambda-Cyhalothrin (15g/l) + Acetamiprid (20g/l) as active ingredient to mitigate aphid infestation at vegetative growth stage; **= systemic and contact Fungicide applied (Carbendazim 12% + Mancozeb 63% W.P.)

#### 2.2.3 Data collection and statistical analysis

Agronomic and phenotypic characteristics (**Table 4**) were recorded for each cowpea entry within all blocks at each site and were subjected to linear mixed model analysis using R, including the “lmerTest” package (Bates, 2014). The mean sample size for each agronomic parameter was five (5) per trial location and a total of 10 for the two locations. For a given agronomic parameter, data were analyzed using the following linear mixed model:

**Table 4:** Description of agronomic and phenotypic parameters.

Characteristic	Description	Growth Stage^1^
Germination percent	Number of plants emerged at 21 days after planting as a percentage of seeds planted.	VE
Days to first flowering	Number of days to the onset of flowering (inflorescence) when there is one open flower per plant.	R1
Days to 50% flowering	Number of days when 50% of the flowers are open.	R2
Plant height	Distance from the soil surface to the base of the top leaf on the main stem (not tendrils). Calculated average value for 20 randomly selected plants per plot and expressed in cm.	R1
Plant vigour	Composite score; 1-5 scale, 1 = relatively small and weak, pale yellow in appearance; 3 = acceptable growth and development; 5 = normal size, strong and erect. Rating is representative of the entire plot.	VE, V1, V3, R1, R2, R3, R7
Days to first pod of maximum length	Assessed on a whole plot basis, the number of day to the first pod of maximum length.	R3
Pods per plant	Number of pods averaged across 20 randomly selected plants per plot.	R8
Pod length	Average length (cm) of up to 20 pods per plant, calculated across 20 randomly selected plants per plot.	R8
*Maruca* damaged pods per plant	Number of damaged pods observed per plant, averaged across 20 randomly selected plants per plot.	R8
Sucking insect damaged pods per plant	Number of damaged pods observed per plant, averaged across 20 randomly selected plants per plot.	R8
Total seed weight per plant	Total weight of seed (g) harvested from each of 20 randomly selected plants per plot, reported as the average (g/plant).	R8
Damaged seed weight per plant	Weight (g) of harvested seed from 20 randomly selected plants per plot exhibiting damage from either *Maruca* pod borer or sucking insects, reported separately.	R8
Hundred seed weight	Weight (g) of 100 seeds from each of 20 randomly selected plants per plot, reported as the average.	R8
Grain Yield	Total weight of seed harvested from the inner two (Nigeria) or three (Ghana) rows of each plot, reported as kg/ha assuming a plant stand of 67,134 plants per hectare.	R8

^1.^Adapted from H.F. Schwartz (Colorado State University) and M.A.C. Langham (South Dakota State University. Available at: https://beanipm.pbgworks.org/cowpea.


$$y_{ijk}=\mu_{i}+l_{j}+r_{k\left(j\right)}+{\left(\mu l\right)}_{ij}+\epsilon_{ijk}$$

Where *μ_i_* denotes the mean of the *i^th^* entry (fixed effect), *l_j_* denotes the effect of the *j^th^* site (fixed effect), *r_k(j)_* denotes the effect of the *k^th^* block within the *j^th^* site (random effect), *(μl)_ij_* denotes the interaction between the entries and sites, and *ϵ_ijk_* denotes the effect of the plot assigned the *i^th^* entry in the *k^th^* block of the *j^th^* site (random effect or residual) (Bates, 2014).

The “lmer” procedure from the “lmerTest” package was used to fit the linear mixed model and to generate estimates of variance components and p-values. For each agronomic parameter, the least square (LS) mean value across sites was estimated from the corresponding statistical model for each entry using the “emmeans” package (Lenth, 2013) where there was no significant site effect.

### 2.3 Post-harvest storage studies

#### 2.3.1 Laboratory rearing of weevils

Cowpea weevils were reared at the Entomology Laboratories of CSIR–SARI and IAR using the methodology described by Swella and Mushobozy (2007). The cowpea seeds used for the rearing were obtained from the Cowpea Improvement Programmes of CSIR–SARI and IAR. These were prepared by freezing at -4°C for 48 h followed by oven drying at 60°C for 24 h. A total of 500 g of the resulting seed were weighed into individual clean Kilner jars followed by infesting the seed with 50 unsexed adult weevils. These adult weevils were obtained from weevil colonies maintained by the research institutes. After seven days, the adults were sieved out and the jars containing cowpeas with eggs were incubated at a temperature of 27 ± 3°C and relative humidity (RH) of 50–70%. At approximately 21 days after infestation (DAI), the newly emerged cowpea weevil cohorts were sieved out and used to infest seed samples for the trial. The peak of female egg laying occurs about 3–4 days after emergence begins and thus, all insects used for the tests were not older than 48 hr.

#### 2.3.2 Storage trial establishment and management

The methodology described by Tengey et al. (2023) with slight modifications was used in this study. Briefly, a total of 250 g of harvested seed from each field trial plot was sterilized by freezing at -4°C for 48 h followed by oven drying at 60°C for 24 h. The oven-dried samples were allowed to cool overnight and then sorted to remove insect and mechanically damaged grains, as well as seeds that had egg deposits on them. Afterwards, 200 g of the clean seed for each test entry were weighed into individual 500 ml Kilner jars. Each jar was labelled with information on the identity of its test entry, plot number, and the field trial block from which it was collected. A total of 100 randomly selected seeds were removed from each jar and weighed to obtain the initial 100-seed weight for each entry. These were returned back to the jars after which 10 randomly selected seeds of CSI-32 were removed from jars of each plot and placed in labelled plastic bags for quantitative analysis of αAI-1 content.

The jars were then infested with 50 adult cowpea weevils (25 males and 25 females) and covered with clean cheesecloth and then tightened with a perforated lid to allow for ventilation. They were then arranged in a completely randomized design (CRD) on a laboratory bench in the trial rooms at the testing Entomology Laboratories. The temperature and RH in these trial rooms were maintained at 27 ± 3°C and 50–70%, respectively.

#### 2.3.3 Data collection and statistical analysis

The storage trial was conducted for a period of 120 days, sufficient for the emergence of four filial generations (F1 through F4) of emerged weevils, during which time the following data were collected and/or computed:

##### 2.3.3.1 Number of eggs per 100 seeds

On the 8^th^ DAI, the adult weevils were sieved out with the aid of a fine-mesh sieve followed by randomly selecting 100 seeds from each entry and counting the number of eggs per seed as described by Lambert et al. (1985). All grains were placed back into their respective jars afterwards, and jars were then returned to the storage trial room.

##### 2.3.3.2 Adult emergence

From the 12^th^ DAI onwards, the experimental setups were examined daily and the days to first adult emergence (DFE) were recorded. Afterwards, the number of weevils emerging in each jar were recorded daily until the n^th^ day after first emergence when there was no change in the numbers counted (i.e., emergence was completed), which marked the end of a generation (F). This process was repeated until completion of the F4 generation. To count weevils, each experimental unit was sieved with a fine mesh sieve into a large container and the sieved weevils were frozen for three to five minutes to immobilize them for easy counting. The immobilized weevils were divided into approximately 10 equal portions and counted three times, after which they were placed back into their respective jars. The mean values were added to obtain the total weevil population.

##### 2.3.3.3 Seed weight loss and percent seed damage

Seed weight loss was measured at the end of the F4 generation by randomly selecting 100 seeds from each entry and recording the weight in comparison to the initial weight measured at the start of the trial. The percent weight loss was calculated using the formula:


$$\begin{array}{c} \text{Percent weight loss }(\%)\\ =\frac{\text{Initial weight (g)}-\text{Final weight (g)}}{\text{Initial weight (g)}}\times 100\% \end{array}$$

At the end of each filial generation, the seeds from each entry were sorted into damaged and undamaged seed, followed by counting the number in each category. Damaged seeds were those that had at least one exit hole on the seed surface while undamaged ones had no exit hole. The percent seed damage was calculated as:


$$\text{Percent seed damaged (\%)}=\frac{\text{Number of damaged seed}}{\text{Total number of seed}}\times 100\%$$

##### 2.3.3.4 Median development period

The MDP is the number of days taken for 50% of the adults to emerge, was calculated using the following formula (Howe, 1971; Nyarko et al., 2022);


$$\text{MDP}=\frac{d_{1}a_{1}+d_{2}a_{2}+d_{3}a_{3}+\ldots +d_{n}a_{n}}{\text{Total Adults Emerged}}$$

Where *d_1_* = day at which adults started emerging and *a_1_* = number of adults emerged on *d_1_^th^* day, and so on until the end of adult emergence (*d_n_a_n_*).

##### 2.3.3.5 Dobie susceptibility index

The DSI (Dobie, 1974; Dobie and Kilminster, 1978) was calculated for each genotype using the number of adults that emerged at the end of the F1 generation and the MDP using the equation:


$$\text{DSI}=\frac{log_{e}\left(\text{Total number of emerged adults}\right)}{\text{MDP}}\times 100$$

The DSI was used in categorizing the cowpea genotypes into resistant or susceptible classes, where 1−5 = resistant; 6−10 = moderately resistant; 11−15 = susceptible; and >16 = highly susceptible (Chakraborty et al., 2015).

Data on adult emergence, percent seed damage, and computed parameters MDP and DSI, were subjected to linear mixed model analysis as previously described (section 2.2.3).

#### 2.3.4 Quantification of αAI-1 content in event CSI-32 seed

Concentrations of αAI-1 protein in seed samples from each replicated plot of event CSI-32 grown at Nyankpala were determined by enzyme linked immunosorbent assay (ELISA) using a mouse monoclonal antibody (MAb) produced against a synthetic peptide sequence (NDIKSVPWDVHDYDG) derived from the β-chain of αAI-1. Seed samples were ground to a crude powder with a mortar and pestle and then homogenized with 50 mM sodium carbonate pH 9.6 at a ratio of 100 mg/ml using a bead mill homogenizer. The crude homogenate was centrifuged (14,000 rpm × 4 min), diluted 1:10,000 with carbonate buffer, and 100 μl aliquots were dispensed into EIA microtiter plate wells (Nunc, MaxiSorp^®^, Thermo Fisher) and incubated overnight at 4°C. For calibration, αAI-1 was purified from CSI-32 seed essentially as described by Marshall and Lauda (1975) and 100 μl volumes of a dilution series ranging from 1–250 ng/ml were dispensed into microtiter plate wells to construct a standard curve. Following plate washing with TBST (Tris buffered saline, 0.05% Tween 20, pH 7.6) and blocking (TBST + 1% bovine serum albumin), bound αAI-1 protein was detected using horseradish peroxidase (HRP)-conjugated MAb DDP2-MM05 followed by substrate development using 1-Step™ Ultra TMB substrate (Thermo Scientific) and measurement of the absorbance at 660 nm.

## 3 Results

### 3.1 Agronomic and phenotypic characteristics

In the combined-sites analysis, there were small but statistically significant differences between many of the entries for most of the agronomic and phenotypic parameters (**Table 5**). Not surprisingly, entries with different germplasm backgrounds exhibited differences in parameters associated with growth and development (e.g., days to flowering and to first pod of maximum length, plant height, pods per plant, and pod length). Insect damage by pod borers and sucking insects was generally similar across the different entries, except for SAMPEA 20-T, which showed significantly reduced numbers of *Maruca* damaged pods and seed. However, a statistically significant increase in sucking insect damaged pods was observed for SAMPEA 20-T relative to the other entries. Except for IT13K-1070-2, which showed higher yield, the grain yields among the remaining four entries were not significantly different.

**Table 5:** Combined-sites analysis of phenotypic data for all entries grown at Nyankpala and Zaria during 2023.

Parameter^1^	IT86D-1010	SAMPEA 7	IT13K-1070-2	SAMPEA 20-T	CSI-32
Germination (%)	77.8^a^ ± 12.7 (50.5–93.8)	82.3^a^ ± 10.9 (59.5–92.8)	76^a^ ± 10.7 (56–86.5)	74.9^a^ ± 14.7 (55–96.6)	81.8^a^ ± 11.1 (57.7–93.8)
Plant Height (cm)^2^	63.6^cd^ ± 17.3 (33.6–91.6)	56.7^bc^ ± 20 (32.3–99.8)	45.6^a^ ± 4.1 (40.1–53.4)	48.6^ab^ ± 9.5 (31–63.4)	67.7^d^ ± 21.3 (31.2–103.2)
Days to First Flowering	38.3^a^ ± 2.7 (35–41)	43.7^c^ ± 5.0 (38–50)	40.4^b^ ± 2.8 (37–44)	38.1^a^ ± 3.1 (34–42)	37.8^a^ ± 2.1 (35–41)
Days to 50% Flowering	43.4^ab^ ± 3.5 (40–49)	49.9^c^ ± 5.2 (44–55)	44^b^ ± 3.0 (41–48)	42.8^a^ ± 3.9 (39–48)	43.1^a^ ± 3.5 (39–47)
Days to First Pod of Maximum Length	49.4^b^ ± 2.3 (47–53)	54.3^d^ ± 1.5 (52–56)	51.3^c^ ± 2.0 (49–54)	49.8^b^ ± 1.8 (47–52)	48.5^a^ ± 1.8 (46–51)
Pods per Plant	13.8^a^ ± 3.2 (9–20.9)	14.0^a^ ± 4.1 (8.4–22.9)	18.5^b^ ± 5.1 (8.6–25.6)	15.9^ab^ ± 3.3 (8.3–19)	15.7^ab^ ± 2.0 (12.1–19.6)
Pod Length (cm)	15.3^a^ ± 0.9 (12.7–16.3)	16.3^b^ ± 0.46 (15.5–16.4)	14.9^a^ ± 0.53 (14.0–15.9)	16.4^b^ ± 1.1 (14.3–18.1)	15.2^a^ ± 0.63 (14.2–16.4)
*Maruca* Damaged Pods per Plant	1.30^b^ ± 0.94 (0.25–3)	0.89^ab^ ± 0.69 (0.2–2.4)	1.46^b^ ± 1.41 (0.3–4.7)	0.10^a^ ± 0.14 (0–0.45)	1.33^b^ ± 1.21 (0.2–3.6)
Sucking Insect Damaged Pods	2.08^a^ ± 0.91 (0.2–3.2)	2.46^ab^ ± 1.13 (1.1–5.4)	3.07^ab^ ± 1.34 (0.5–6.1)	3.47^b^ ± 1.69 (0.4–6.1)	2.75^ab^ ± 1.03 (0.9–4.3)
*Maruca* Damaged Seed Weight (g/plant)	0.15^ab^ ± 0.07 (0.08–0.32)	0.19^b^ ± 0.11 (0.01–0.33)	0.27^b^ ± 0.19 (0.1–0.78)	0.04^a^ ± 0.05 (0–0.1)	0.16^ab^ ± 0.15 (0.05–0.53)
Sucking Insect Damaged Seed Weight (g/plant)	0.77^a^ ± 0.58 (0.21–2.07)	2.45^b^ ± 1.74 (0.58–4.94)	1.36^a^ ± 1.12 (0.2–3.82)	0.87^a^ ± 0.43 (0.11–1.47)	0.88^a^ ± 0.77 (0.12–2.26)
Healthy Seed Weight (g/plant)	15.3^b^ ± 4.5 (11.2–26.5)	10.4^a^ ± 4.9 (1.7–18.2)	18.5^b^ ± 6.5 (9.8–29)	14.6^ab^ ± 2.9 (8.4–17.8)	17.6^b^ ± 3.7 (13.6–25.4)
Hundred Seed Weight (g)	15.4^b^ ± 1.6 (14–20)	15.7^b^ ± 1.0 (14–17)	13.1^a^ ± 1.0 (12–16)	18.8^c^ ± 0.8 (17–19.5)	14.8^b^ ± 1.0 (14–17)
Grain Yield (kg/ha)	696^a^ ± 128 (540–902)	730^a^ ± 290 (440–1335)	997^b^ ± 105 (796–1162)	718^a^ ± 82 (595–861)	651^a^ ± 106 (506–806)

^1.^Values for each parameter represent the least square (LS) means from six replicated blocks of each entry grown at Nyankpala and Zaria during 2023 (N=12). For each parameter, the range of values is shown in parentheses. Data were subjected to linear mixed model analysis with genotype and location as the fixed effects to generate LS means and estimates of statistical significance for any differences (p<0.05).

^1.^For each parameter across entries, LS mean values with the same superscript are not significantly different.

In direct comparisons between event CSI-32 and its non-transformed conventional counterpart, IT86D-1010, the only statistically significant differences were the number of days to first pod of maximum length (FPML), which was approximately one day shorter for CSI-32 cowpea, and the number of sucking insect damaged pods per plant, which were 2.75 for event CSI-32 and 2.08 for the control line (**Table 6**). There were no differences noted between event CSI-32 and control IT86D-1010 cowpea with respect to plant vigour at either trial site, and at both locations, the transgenic and control entries exhibited normal growth and development with no significant differences in grain yield.

**Table 6:** Combined sites analysis of phenotypic data for CSI-32 and control cowpea grown at Nyankpala and Zaria during 2023.

Entries	Germination (%)^1^	Flowering (days)		Plant Height (cm)	Pods		
		First Flowering	50% Flowering		FPML^2^ (days)	Count/Plant	Length (cm)
CSI-32	81.8 ± 11.1 (57.7–93.8)	37.8 ± 2.1 (35–41)	43.1 ± 3.5 (39–47)	67.7 ± 21.3 (31.2–103)	48.5 ± 1.8 (46–51)	15.7 ± 2.0 (12.1–19.6)	15.2 ± 0.6 (14.2–16.4)
IT86D-1010	77.8 ± 12.7 (50.5–93.8)	38.3 ± 2.7 (35–41)	43.4 ± 3.5 (40–49)	63.6 ± 17.3 (33.6–91.6)	49.4 ± 2.3 (47–53)	13.8 ± 3.2 (9–20.9)	15.3 ± 0.9 (12.7–16.3)
p-Values (genotype)	0.342	0.179	0.358	0.066	*0.006*	0.099	0.665

Entries	*Maruca* Damage		Sucking Insect Damage				
	Pods/Plant	Seed (g/plant)	Pods/Plant	Seed (g/plant)		Grain Yield (kg/ha)	100-Seed Weight (g)
CSI-32	1.33 ± 1.21 (0.2–3.6)	0.16 ± 0.15 (0.05–0.53)	2.75 ± 1.03 (0.9–4.3)	0.88 ± 0.77 (0.12–2.26)		651 ± 106 (506–806)	14.8 ± 1.0 (14–17)
IT86D-1010	1.30 ± 0.94 (0.25–3)	0.15 ± 0.07 (0.08–0.32)	2.08 ± 0.91 (0.2–3.2)	0.77 ± 0.58 (0.21–2.07)		696 ± 128 (540–902)	15.4 ± 1.6 (14–20)
p-Values (genotype)	0.901	0.717	***0.016***	0.503		0.234	0.251

^1.^Values represent least square (LS) means of six replicate measurements from event CSI-32 and control IT86D-1010 plants grown at each location in 2023. For each parameter, the range of measured values is shown in parentheses. Data were subjected to linear mixed model analysis with genotype and location as the fixed effects to generate LS means and estimates of statistical significance for any differences (p< 0.05).

^2.^FPML = First pod of maximum length, in days.

### 3.2 Post-harvest storage trial

Bruchid emergence data were collected over four filial generations of cowpea weevils that were observed for every entry except event CSI-32. As evidenced from the combined sites analysis of F1 generation data (**Table 7**), there were no significant differences between IT86D-1010, SAMPEA 7, SAMPEA 20-T, or IT13K-1070-2 in days to first emergence (p=0.065), number of emerged adults (p=0.413), Dobie’s susceptibility index (p=0.651), and the percentage of damaged seed (p=0.434). While there were small, statistically significant differences between the four susceptible entries in median development period (p=0.009), the maximum difference between mean MDP values was only 0.5 days (*ca*. 2%). In the combined sites analysis, all four susceptible varieties were classified as highly susceptible based on mean DSI values >20 (**Table 7**).

**Table 7:** Combined-sites analysis of Bruchid emergence parameters for the F1 generation.

Parameter^1^	IT86D-1010	SAMPEA 7	IT13K-1070-2	SAMPEA 20-T	CSI-32
Eggs Laid^2^ (count/100-seeds)	51^ab^ ± 26.4 (10–89)	64.2^ab^ ± 20.8 (20–90)	52^ab^ ± 31.5 (10–120)	69.1^b^ ± 38.3 (20–130)	37.3^a^ ± 23.7 (10–80)
Days to First Emergence	22.4^a^ ± 1.6 (20–24)	22.2^a^ ± 1.0 (20–23)	22.4^a^ ± 1.4 (20–24)	21.8^a^ ± 1.7 (19–24)	NA
Emerged Adults (count)	257.2^a^ ± 97.5 (111–417)	305.2^a^ ± 144 (90–576)	322.6^a^ ± 202 (16–622)	295.6^a^ ± 139 (91–483)	0^b^ (0–0)
Median Development Period (days)	27.2^b^ ± 1.4 (25.6–29)	26.8^a^ ± 1.5 (25.1–28.9)	27.0^ab^ ± 1.5 (25.3–28.9)	26.7^a^ ± 1.2 (25.1–28.5)	NA
Dobie’s Susceptibility Index	20.1^a^ ± 1.1 (18.4–22)	20.9^a^ ± 1.7 (17.5–23.2)	20.2^a^ ± 3.3 (11.0–23.4)	20.1^a^ ± 2.2 (17.7–23.3)	NA
Damaged Seed (%)	23.3^a^ ± 9.2 (10–39)	22.5^a^ ± 10.7 (5–38)	21.3^a^ ± 10.6 (4–37)	27.3^a^ ± 14.9 (10–56)	0^b^ (0–0)

^1.^Values for each parameter represent the least square (LS) means of six replicate samples from each entry tested at each location (N=12). For each parameter, the range of values is shown in parentheses. Data were subjected to linear mixed model analysis with genotype and location as the fixed effects to generate LS means and estimates of statistical significance for any differences (p<0.05).

^2.^For each parameter, LS mean values with the same superscript at not significantly different.

Event CSI-32, with an average αAI-1 protein concentration of 21.0 ± 2.8 μg/mg in the seed as measured by quantitative ELISA of samples at Nyankpala, exhibited complete suppression of adult weevil emergence and absence of seed damage for the four-month duration of the study (120 days post initial infestation).

**Figure 2:**
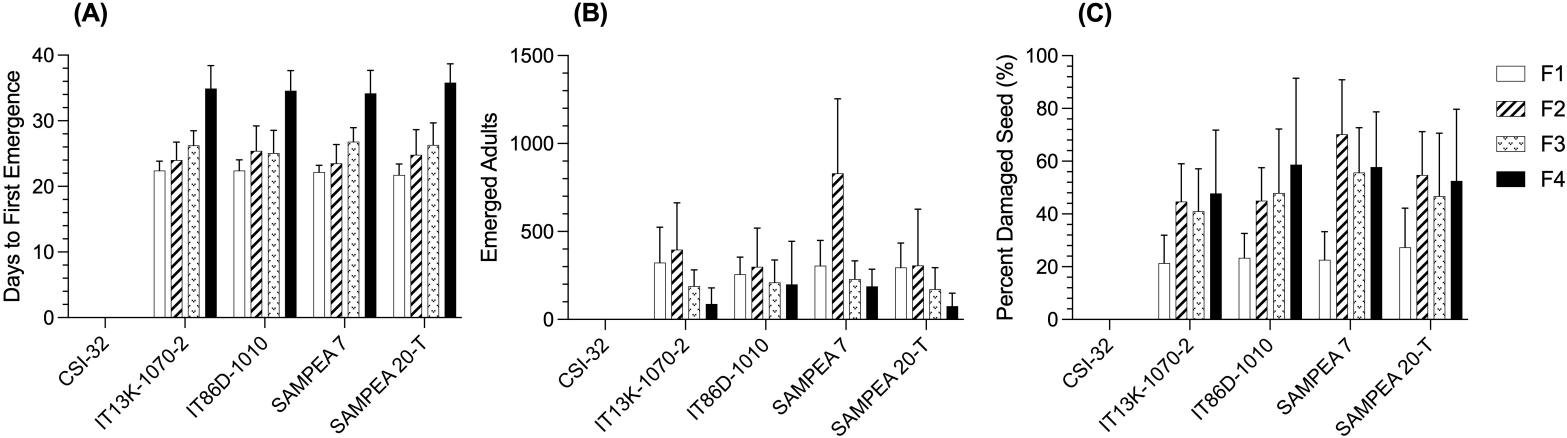
Comparisons of days to first emergence **(A)**, numbers of emerged adults **(B)**, and percent damaged seed **(C)** across the four filial generations (F1–F4) of emerged weevils for each of the test entries, CSI-32, IT13K-1070-2, IT86D-1010, SAMPEA 7, and SAMPEA 20-T. Bars represent least square (LS) mean values of six replicate samples from each location (N=12) and error bars are standard deviations. In the combined sites analysis, data were subjected to linear mixed model analysis with genotype and location as the fixed effects.

The days to first emergence increased with each generation (**Figure 2A**). The days to first emergence for the F4 generation was approximately 12 days later than for the F1 generation and this was not significantly different between the four susceptible cowpea varieties (p=0.391). The number of emerged adults was greatest for the F2 generation (**Figure 2B**) and declined in subsequent generations. The percentage of damaged seed increased with each generation of emerged weevils (**Figure 2C**). After the F4 generation, the percentage of damaged seeds was 43.4 percent, on average, with no significant differences between susceptible varieties (p=0.117).

## 4 Discussion

Genetic variability among cowpea genotypes is crucial for the efficiency of cowpea improvements (Gerrano et al., 2015). These genetic differences account for observed variations in traits like plant height, flowering time, seeds per pod, productive branches, and seed yield (Shimelis and Shiringani, 2010). Therefore, the agronomic and phenotypic differences seen in the studied genotypes are expected. Similar variations in phenotypic traits among genetically diverse cowpea varieties are well documented (Animasaun et al., 2015; Agyeman et al., 2015; Belay et al., 2017; Nkoana et al., 2019).

The transgenic event, CSI-32, and its near-isogenic conventional comparator, IT86D-1010 exhibited normal growth and development when phenotypic parameters, including plant vigor were measured. The only difference detected between line CSI-32 and the control IT86D-1010 was the resistance to bruchid damage, which was the goal for the introduced trait. The findings suggest that the introduced *αAI-1* gene and production of high levels of αAI-1 protein in the seed of transgenic event CSI-32 did not give rise to unintended phenotypic effects. Similar reports of a lack of significant difference in phenotypic characters between the transgenic Cry1Ab-expressing event, SAMPEA 20-T, and control cowpea entries were reported by Addae et al. (2020).

Damage to cowpea by the pod-sucking bug (PSB) complex (*C. tomentosicollis*, *A. curvipes*, *R dentipes*, *N. viridula*, *T. custator* and *A. armigera*) did not differ among the entries. To date, cowpea breeding programmes have not succeeded in developing varieties that are stably resistant to multiple damaging pests of the crop, including PSBs (Togola et al., 2017). This is because several minor genes (polygenic resistance) regulate the control of multiple insect pests’ attacks. Until recently, the introgression of these minor genes into new varieties was considered complex. In recent times however, advances in biotechnology makes it possible to introgress these genes into improved cowpea varieties (Sharma et al., 2002; Kogan, 1994). This notwithstanding, none of the cowpea entries used in this work were resistant to the PSB pest complex. Damage by these sucking pests probably accounted for the differences in the 100-seed weight at Nyankpala in particular.

Apart from SAMPEA 20-T, which expresses the Cry1Ab insecticidal protein conferring resistance to damage by *Maruca* pod borer (Addae et al., 2020), all the genotypes, including CSI-32, were susceptible to damage by this pest. The expression of αAI-1 protein in CSI-32 seed would not be expected to confer protection against pod borers and the in-field pest observations were consistent with the activity spectrum of αAI-1.

Cowpea yields are impacted by environmental variables (rainfall, temperature), genotypic potential, and pests and diseases (Cerıtoglu and Erman, 2020; Jackai and Daoust, 1986; Ibrahim et al., 2021; Karungi et al., 2000). In this study, a uniform spray regime was applied to all entries to control arthropod pests, and their exposure to elements of the weather were the same. Hence, grain yield was determined largely by the yield potential of the individual genotypes assessed. Insect damage did not significantly affect yield. Although genotype was a significant factor for yield (p<0.001), yields were very similar among four of the five entries, with only IT13K-1070-2 exhibiting higher yield at both locations.

Except landraces (Silva et al., 2021), many studies that have evaluated commercially cultivated improved cowpea varieties for weevil resistance, have been unsuccessful in identifying durable sources of resistance to this voracious pest (Badii et al., 2013; Deshpande et al., 2011; Kpoviessi et al., 2019; Amusa et al., 2018). Similarly, none of the commercial cowpea entries tested in this work were resistant to *C. maculatus*. The genotype, SAMPEA 7, which is reported to be susceptible to weevils (Azeez and Pitan, 2013; Ojumoola and Adesiyun, 2014; Ofuya and Credland, 1995) and IT13K-1070-2, which is reported to be moderately resistant to this pest (Tengey et al., 2023), were both found to be highly susceptible to weevils when they were subjected to an infestation pressure of 50 sexually matured insects per 200-g seed sample. Oviposition was similar across all the entries tested, including CSI-32, indicating that αAI-1 did not act as a deterrent to oviposition; however, it did act to completely suppress larval development and adult emergence during the 4-month duration of the study.

The median development period (MDP), adult emergence, and severity of grain damage are important determinants of ranking cowpea genotypes as being susceptible to cowpea weevils. Prolonged development time, low adult emergence, and low grain damage are key characteristics of resistant genotypes and vice versa (Amusa et al., 2018; Azeez and Pitan, 2013; Silva et al., 2021). Additionally, Badii et al. (2013) and Amusa et al. (2018) reported a difference in MDP of *ca.* 3 and *ca* 6 days, respectively, between susceptible and resistant cowpea genotypes. Here, we report *ca.* 0.5 days difference between the highest and lowest MDP, which is unlikely to be biologically meaningful. The genotypes, IT86D-1010, SAMPEA-7, IT13K-1070-2 and SAMPEA 20-T, were categorized as being highly susceptible based on mean Dobie’s Susceptibility Index (DSI) values >20 for each entry. Further, the survival and emergence of adults as well as the overall high levels of seed damage recorded for these genotypes confirmed their susceptibility.

This is the first study to examine both in-field agronomic performance and post-harvest storability of a transgenic cowpea expressing αAI-1 to control the cowpea weevil. The findings of the current study and earlier ones clearly demonstrate that developing cowpea varieties that express the αAI-1 protein presents a sustainable and cost-effective approach to mitigating damage by this pest. The technology to control the weevil is incorporated in the seed itself and protection is not reliant on the application of synthetic insecticides that have known adverse health and environmental consequences (Kolawole et al., 2014; Kpoviessi et al., 2019). Further, this technology could complement the use of hermetic sealed bags, especially for those who can afford the bags in Africa (Hajam and Kumar, 2022; Silva et al., 2018).

Genetic modification approaches have proven highly effective at separately controlling key field pests, such as *M. vitrata*, and as shown here, the post-harvest storage pest, *C. maculatus*. As discussed in Barrero et al. (2021), future work could include the development of novel cowpea varieties combining these two important insect resistance traits for the benefit of smallholder farmers and consumers in West Africa.

## Data Availability

The raw data supporting the conclusions of this article will be made available by the authors, without undue reservation.
